# Oxidative stress, apoptosis and inflammatory responses involved in copper-induced pulmonary toxicity in mice

**DOI:** 10.18632/aging.103585

**Published:** 2020-07-04

**Authors:** Zhijie Jian, Hongrui Guo, Huan Liu, Hengmin Cui, Jing Fang, Zhicai Zuo, Junliang Deng, Yinglun Li, Xun Wang, Ling Zhao

**Affiliations:** 1 College of Veterinary Medicine, Sichuan Agricultural University, Wenjiang 611130, Chengdu, China; 2 Key Laboratory of Animal Diseases and Environmental Hazards of Sichuan Province, Sichuan Agriculture University, Wenjiang 611130, Chengdu, China; 3 Key Laboratory of Agricultural Information Engineering of Sichuan Province, Sichuan Agriculture University, Yaan 625014, Sichuan, China

**Keywords:** CuSO _4_, lung, oxidative stress, apoptosis, inflammation, mice

## Abstract

At present, there are few studies focused on the relationship between copper (Cu) and oxidative stress, apoptosis, or inflammatory responses in animal and human lungs. This study was conducted to explore the effects of Cu on pulmonary oxidative stress, apoptosis and inflammatory responses in mice orally administered with 0 mg/kg (control), 10 mg/kg, 20 mg/kg, and 40 mg/kg of CuSO_4_ for 42 days. The results showed that CuSO_4_ increased ROS production, and MDA, 8-OHdG and NO contents as well as iNOS activities and mRNA expression levels. Meanwhile, CuSO_4_ reduced the activities and mRNA expression levels of antioxidant enzymes (GSH-Px, CAT, and SOD) and GSH contents, and ASA and AHR abilities. Also, CuSO_4_ induced apoptosis, which was accompanied by decreasing Bcl-2, Bcl-xL mRNA expression levels and protein expression levels, and increasing Bax, Bak, cleaved-caspase-3, cleaved-caspase-9 mRNA, and protein expression levels, and Bax/Bcl-2 ratio. Concurrently, CuSO_4_ caused inflammation by increasing MPO activities and activating the NF-κB signalling pathway, and down-regulating the mRNA and protein expression levels of anti-inflammatory cytokines (IL-2, IL-4, IL-10). In conclusion, the abovementioned findings demonstrated that over 10 mg/kg CuSO_4_ can cause oxidative stress, apoptosis, and inflammatory responses, which contribute to pulmonary lesions and dysfunction in mice.

## INTRODUCTION

Copper (Cu), as an essential element, is the main metal used in bronze and brass [1–3]. It plays an indispensable role in both animals and plants [4] and also is widely used in pipes, wires, cooking utensils, ornaments and jewellery [5, 6]. Wide industrial use of Cu leads to increased copper pollution in the environment, enhancing the risk of copper toxicity in humans and animals [7–10]. Usually, workers who are continuously exposed to copper face high risk of lung damage, and lower respiratory function is associated with higher serum copper concentrations [11, 12]. It has been reported that oral overdose of copper sulphate (CuSO_4_) can repress body development and impair organic function [13]. Wistar rats fed on diets contaminated with high CuSO_4_ levels exhibit growth depression [14].

Oxidative stress results from an imbalance between the oxidative and antioxidant systems of cells and tissues, and is the result of excessive production of oxidative free radicals and related reactive oxygen species (ROS) [15]. It has been identified that high Cu levels have toxic effects including ROS generation leading to DNA damage [16], oxidative damage to biological molecules [17], and peroxidation of cell membrane lipids [18]. In addition, studies have shown that Cu overload leads to oxidative stress, and subsequently damages proteins, lipids and nucleic acids [19–23]. *In vivo* studies, high dietary copper can inhibit the serum, hepatic [24–27] and nephritic [28] antioxidase activities in ducklings, and induce oxidative stress in the brain [29, 30] and spleen [31] of chickens. Also, it has been reported that intradermal injection of 2% CuSO_4_ solution in the chicken can increase oxygen-derived free radicals, result in tissue damage and increase vascular permeability [32]. In *in vitro* studies, Cu and Cu compounds can cause oxidative stress via the production of ROS in human epithelial lung (A549) cells [33] and brain microvascular endothelial cells [34].

Additionally, increased intracellular ROS as a signal of oxidative stress, can break single or double strands of DNA and activate DNA-dependent protein kinase, leading to apoptosis [35, 36]. Reports of *in vivo* studies have shown that high Cu levels induce the higher percentage of apoptotic cells of the lymphoid organs [37] and kidney [38] in ducklings and lymphoid organs [39], kidney [40], and liver [41] in chickens. The findings of Guo et al. (2017) indicated that Cu can induce oxidative stress and apoptosis in the White Shrimp (*Litopenaeus vannamei)* [42]. Besides, it has been reported that Cu induces apoptosis by increasing the expression levels of cysteine aspartate specific protease-3(caspase-3), cysteinyl aspartate-specific protease9 (caspase-9), and Bcl-2 associated X protein(Bax) in the rat kidney and liver [43, 44]. In *in vitro* studies, the findings have indicated that Cu can induce mitochondrial dysfunction in primary culture of chicken hepatocytes [45] and apoptosis in human skin melanoma A-375 cell line [46] and Aedes C6/36 cells [47].

Inflammation is a defensive response to stimulation and usually is beneficial for animals and human beings [48]: however, continued inflammation can cause damage to the body [49]. Inflammation can be triggered by a variety of factors, such as pathogens, damaged cells, toxic compounds, and irradiation [50]. In addition, oxidative damage can lead to cell metabolism disorders, which accelerates the production of inflammation [51]. In *in vivo* studies, the study has investigated the potential synergistic effects of chronic exposure to Cu in promoting inflammatory and oxidative events in mouse brains [52]. Brand et al. (2019) has found that inhalation of fumes containing Cu can induce asymptomatic systemic inflammation [53]. Pharyngeal aspiration of CuO nanoparticles causes lung inflammation and lung injuries in Wistar rats [54], however, no reports are focused on CuSO_4_-induced inflammation in the human and animal lung.

The aforementioned studies suggest that oxidative stress, apoptosis, and inflammatory responses are the three main mechanisms involved in Cu-induced toxic effects on organs, tissues and cells, however, there are few studies focused on the relationship between Cu and oxidative stress, apoptosis, inflammatory responses in animal and human lungs. Therefore, the present study aims to assess pulmonary toxicity via oxidative stress, apoptosis and inflammatory responses after CuSO_4_ intake in mice by using methods of histopathology, qRT-PCR, and ELISA, flow cytometry, and western blot assay.

## RESULTS

### Histopathological changes in the lung

**Figure 1:**
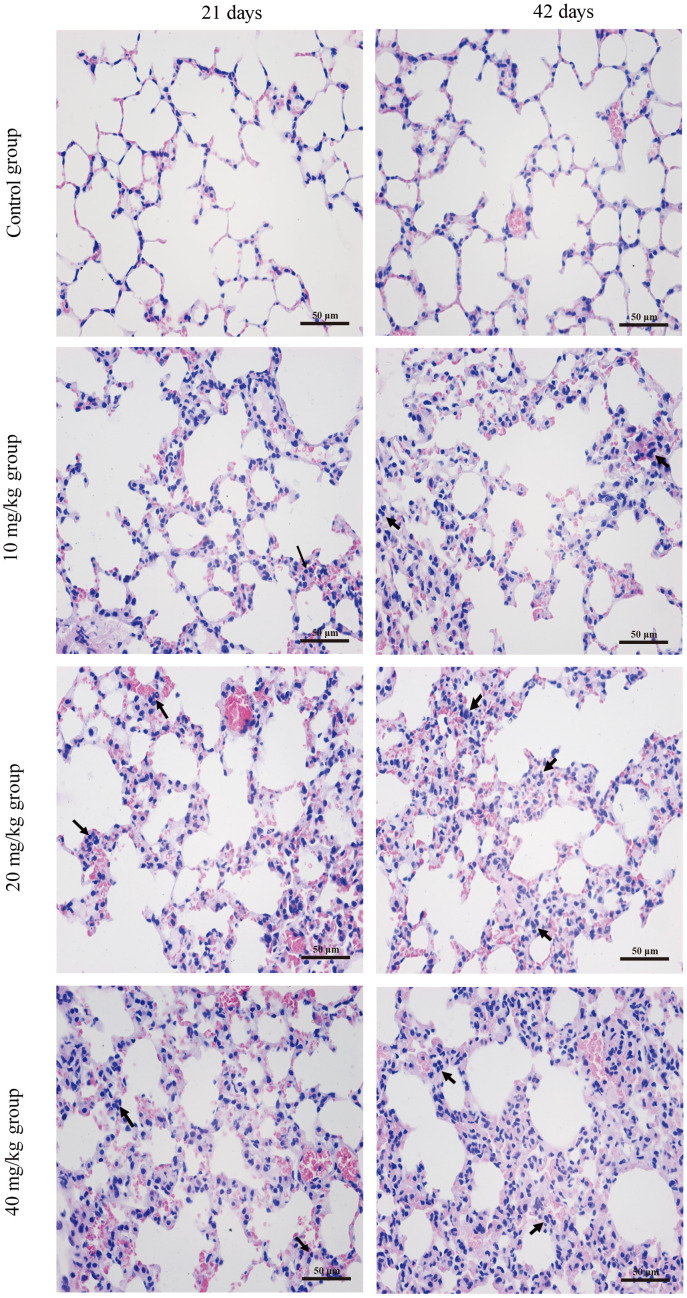
**Histopathological changes in the lung at 21 and 42 days of the experiment. (H&E ×400).** Control group at 21 and 42 days: no changes are observed. 10 mg/kg group at 21 days: the inflammatory cells (↑) are obviously observed in alveolar walls. 20 mg/kg group at 21 days: the alveolar walls are slightly thickened with inflammatory cell infiltration (↑) 40 mg/kg group at 21 days: the alveolar walls are obviously thickened with inflammatory cell infiltration (↑). 10 mg/kg group at 42 days: the alveolar walls are slightly thickened with inflammatory cell infiltration (↑). 20 mg/kg group at 42 days: the alveolar walls are thickened with inflammatory cell infiltration (↑). 40 mg/kg group at 42 days: the alveolar walls are markedly thickened with inflammatory cell infiltration (↑).

Figure 1 shows the dose- and time-dependent lesions in lungs at the three CuSO_4_-treated groups. Histopathologically, alveolar walls were thickened in varying degrees, mainly due to inflammatory cells infiltration and capillary congestion. The aforementioned histopathological lesions were not observed in the control group.

### Changes of ROS production levels and Cu contents in the lung

**Figure 2:**
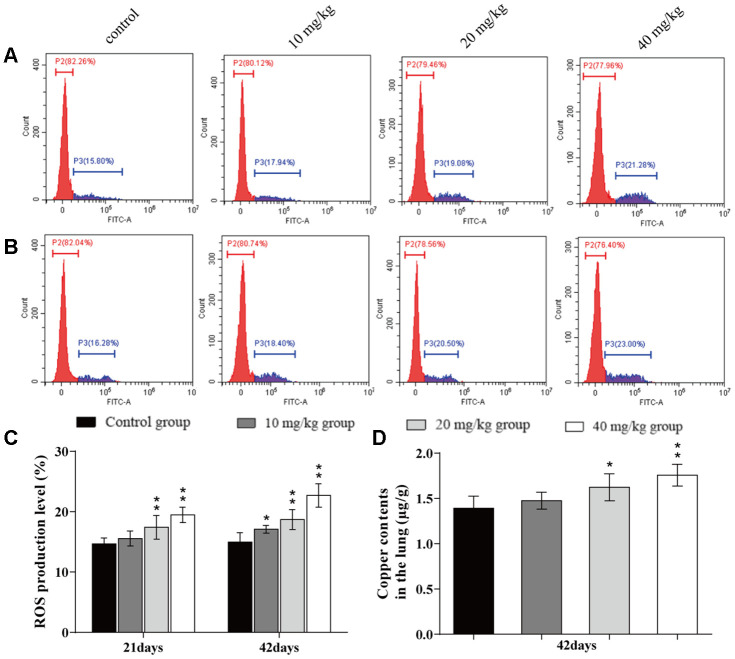
**Changes of ROS production levels and Cu contents in the lung.** (**A**) ROS production levels in the lung by flow cytometry at 21 days of the experiment. (**B**) ROS production levels in the lung by flow cytometry at 42 days. (**C**) ROS production levels in the lung. (**D**) Cu contents in the lung at 42 days of the experiment. Data are presented with the mean± standard deviation (n=8). *p < 0.05, compared with the control group; **p < 0.01, compared with the control group.

The ROS production levels were increased (*p* < 0.05 or *p* < 0.01) in the 20 and 40 mg/kg groups at day 21 of the experiment and in the three CuSO_4_-treated groups at day 42 of the experiment when compared to the control group. The Cu contents in the lung were increased (*p* < 0.05 or *p* < 0.01) in the 20 and 40 mg/kg group at day 42 of the experiment when compared to the control group. The results are illustrated in Figure 2.

### Changes of factors related to ROS production in the lung

The contents of malondialdehyde (MDA) and 8-hydroxy-2'-deoxyguanosine (8-OHdG) were significantly higher (*p* < 0.05 or *p* < 0.01) in the three CuSO_4_-treated groups at day 21 and 42 of the experiment than in the control group.

The anti-superoxide anion (ASA) and anti-hydroxyl radical (AHR) abilities were significantly decreased (*p* < 0.05 or *p* < 0.01) in the 20 and 40 mg/kg groups at day 21 of the experiment and in the three CuSO_4_-treated groups at day 42 of the experiment when compared to the control group.

**Figure 3:**
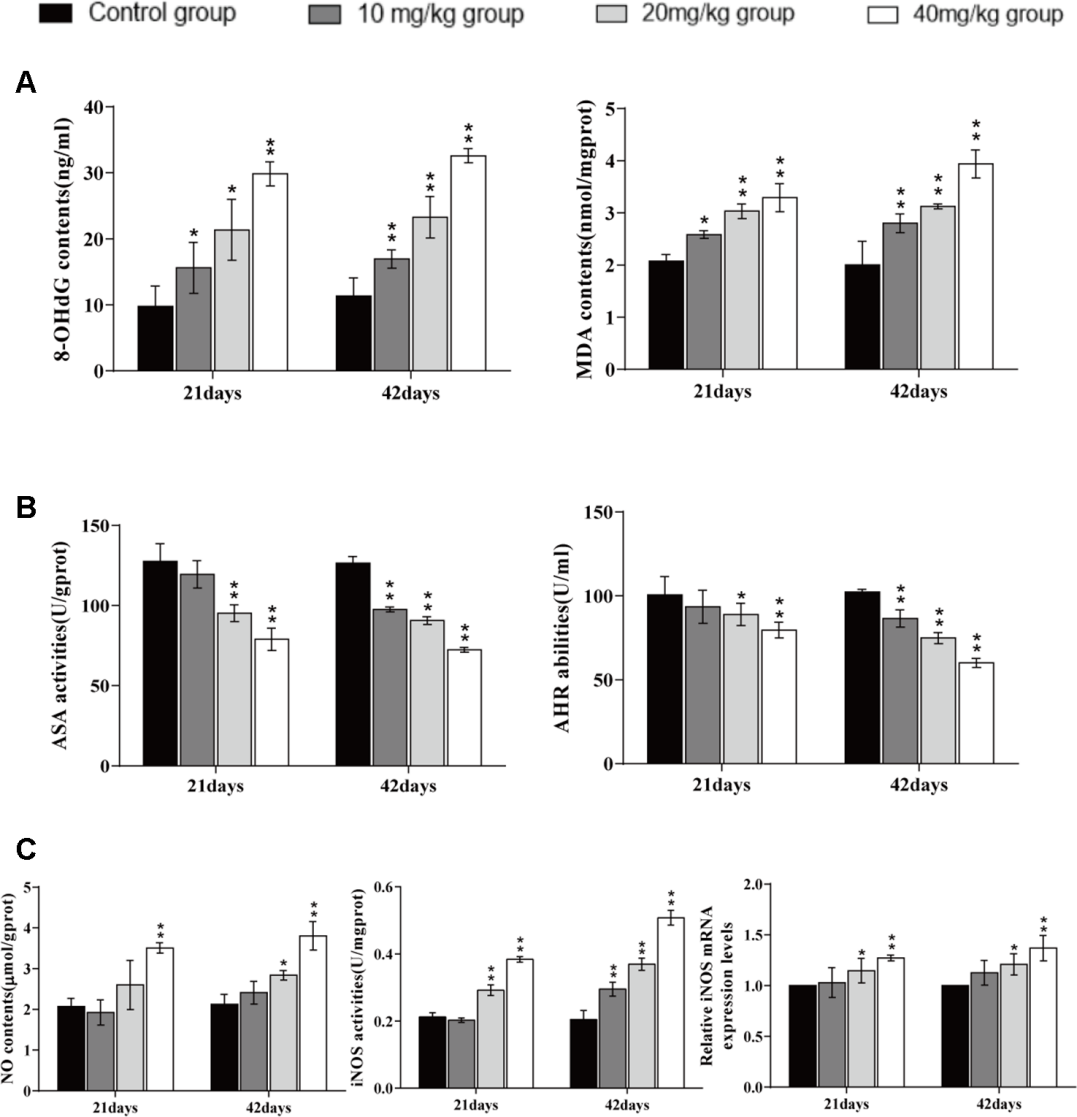
**Changes of factors related to ROS production in the lung.** (**A**). 8-OHdG and MDA contents levels in the lung at 21 and 42 days of the experiment. (**B**) ASA and AHR activities in the lung at 21 and 42 days of the experiment. (**C**) NO contents, iNOS activities, and mRNA expression in the lung at 21 and 42 days of the experiment. Data are presented with the mean± standard deviation (n=8). *p < 0.05, compared with the control group; **p < 0.01, compared with the control group.

The nitric oxide (NO) content was significantly higher (*p* < 0.05 or *p* < 0.01) in the 40 mg/kg group at day 21 of the experiment and in the 20 and 40 mg/kg groups at day 42 of the experiment than in the control group. The inducible nitric oxide synthase (iNOS) activities and mRNA expression levels were significantly increased (*p* < 0.05 or *p* < 0.01) in the 20 and 40 mg/kg groups at day 21 and 42 of experiment in comparison to the control group. The results are shown in Figure 3.

### Changes of oxidative damage parameters in the lung

**Figure 4:**
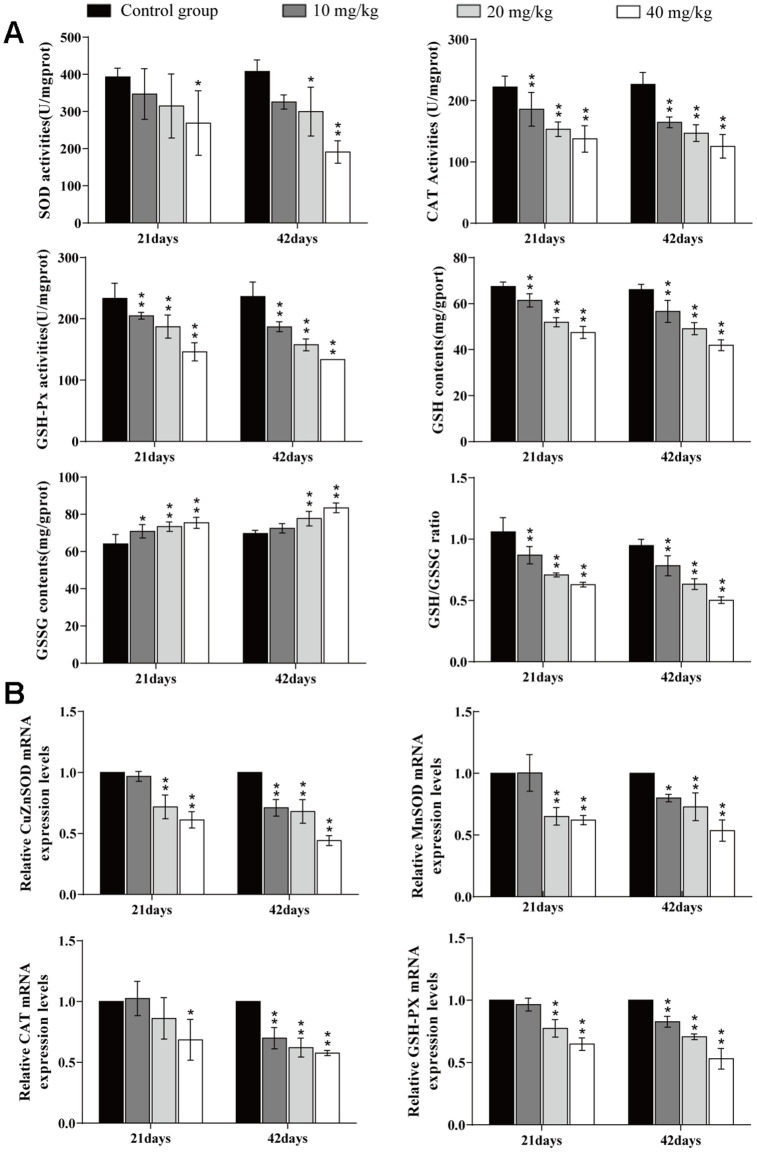
**Changes of oxidative damage parameters in the lung.** (**A**) Changes of antioxidant enzyme activities, GSH and GSSG contents, and GSG/GSSG ratio in the lung at 21 and 42 days of the experiment. (**B**) Changes of mRNA expression levels of antioxidant enzymes in the lung. Data are presented with the mean± standard deviation (n=8). *p < 0.05, compared with the control group; **p < 0.01, compared with the control group.

The superoxide dismutase (SOD) activities were significantly reduced (*p* < 0.05 or *p* < 0.01) in the 40 mg/kg group at 21 and 42 days of the experiment and in the 20 mg/kg group at 42 days in comparison to the control group. The activities of Catalase (CAT), glutathione peroxidase (GSH-Px), Glutathione (GSH) contents, and the GSH/GSSG ratio were significantly decreased (*p* < 0.01) in the three CuSO_4_-treated groups at 21 and 42 days. The oxidised glutathione (GSSG) content was significantly increased (*p* < 0.05 or *p* < 0.01) in the 20 and 40 mg/kg groups at days 21 and 42. The mRNA expression levels of antioxidant enzymes (GSH-Px, CAT, MnSOD, and ZnSOD) were significantly lower (*p* < 0.05 or *p* < 0.01) in the 40 mg/kg group at day 21 and in the three CuSO_4_-treated groups at day 42 than in the control group. The results are shown in Figure 4.

### Changes of apoptosis percentages in the lung

**Figure 5:**
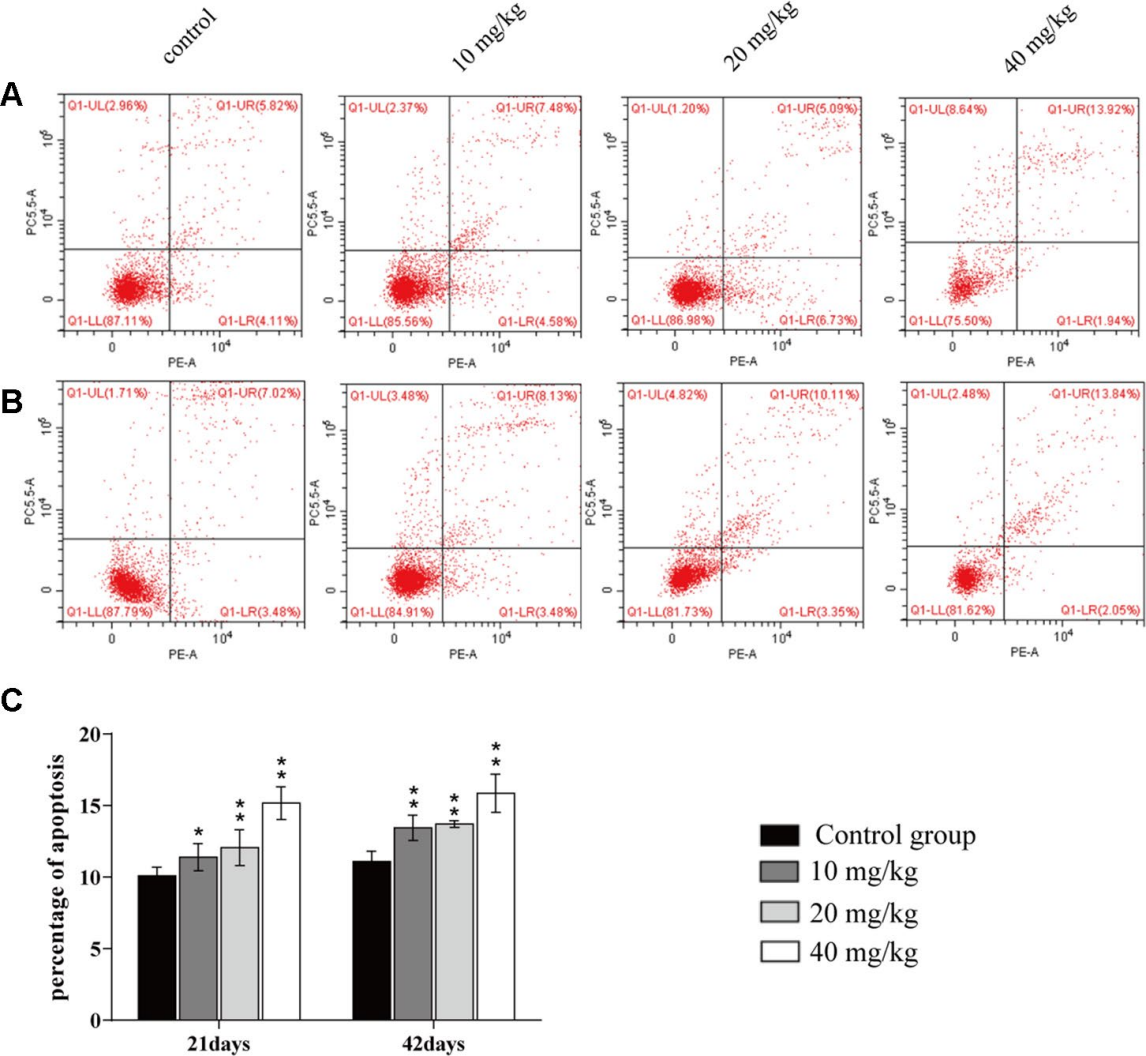
**Changes of apoptosis percentages in the lung.** (**A**) Apoptosis in the lung at 21 days of the experiment. (**B**) Apoptosis in the lung at 42 days. (**C**) Percentage of apoptosis in the lung. Data are presented with the mean± standard deviation (n=8). *p < 0.05, compared with the control group; **p < 0.01, compared with the control group.

The apoptotic percentages were elevated (*p* < 0.05 or *p* < 0.01) in the three CuSO_4_-treated groups at days 21 and 42 of the experiment when compared to the control group, as shown in Figure 5.

### Changes of mRNA and protein expression levels of apoptosis-related mediators in the lung

**Figure 6:**
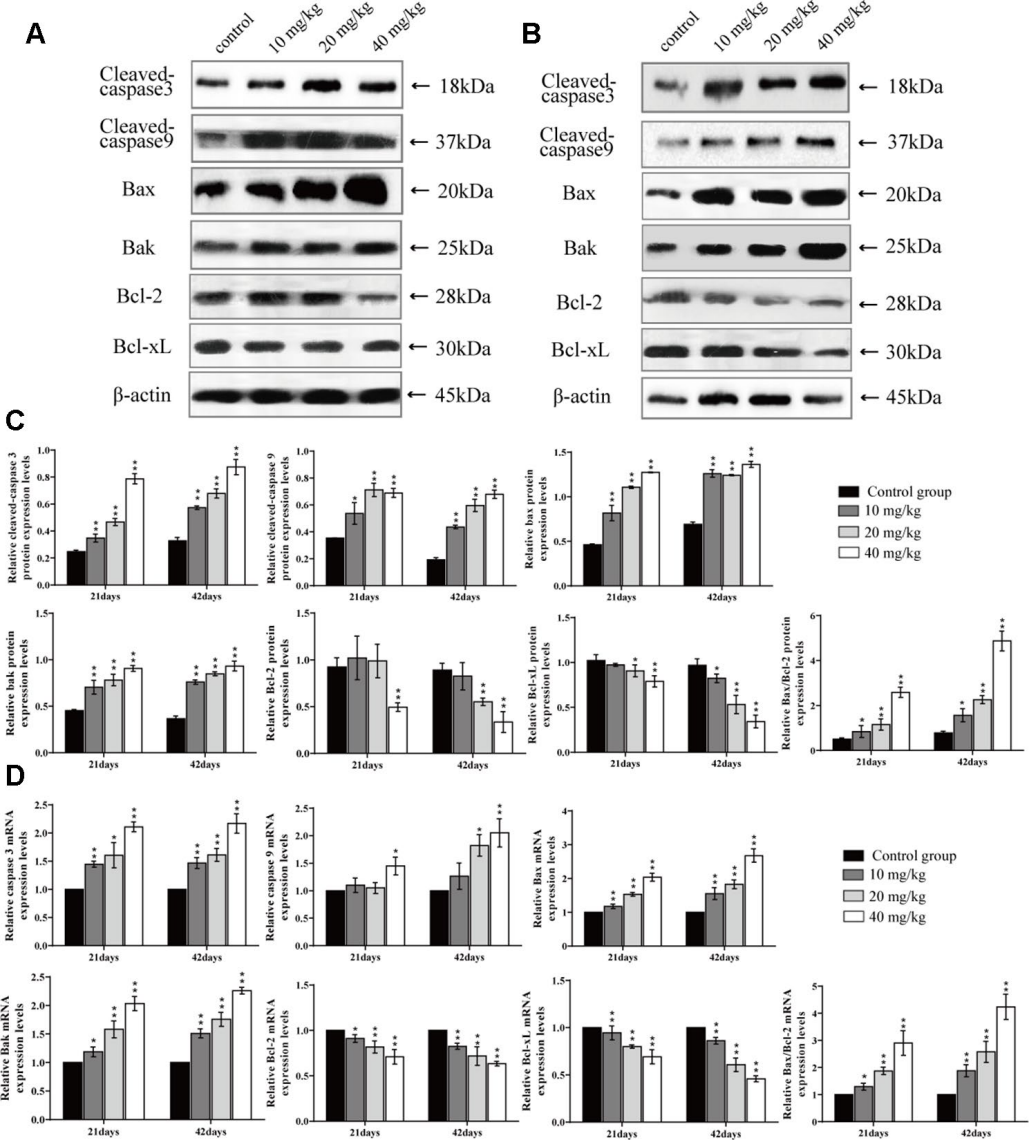
**Changes of protein and mRNA expression levels of apoptotic related mediators in the lung at 21 and 42 days of the experiment.** (**A**) Western blot assay of apoptosis-related mediators at 21 days. (**B**) Western blot assay of apoptosis-related mediators at 42 days. (**C**) The relative protein expression levels of apoptosis-related mediators. (**D**) The relative mRNA expression levels of apoptosis-related mediators. Data are presented with the mean± standard deviation (n=8). *p < 0.05, compared with the control group; **p < 0.01, compared with the control group.

The mRNA expression levels of caspase 3, Bax, Bcl-2 antagonist killer (Bak), and Bax/BcL-2, and the protein expression levels of cleaved-caspase 3, cleaved-caspase 9, Bax, Bak, and Bax/BcL-2 were significantly higher (*p* < 0.01 or *p* <0.05) in the three CuSO_4_-treated groups at 21 and 42 days of the experiment than in the control group. The mRNA expression levels of the B-cell lymphoma-2 (Bcl-2) and Bcl-extra-large (Bcl-xL) were significantly decreased (*p* < 0.01 or *p* < 0.05) in the three CuSO_4_-treated groups at 21 and 42 days when compared to the control group. Also, protein expression levels of Bcl-2 and Bcl-xL were significantly decreased (*p* < 0.01) in the 40 mg/kg group at 21 days and in the 20 and 40 mg/kg groups at 42 days in comparison to the control group, as shown in Figure 6.

### Changes of MPO activities and PGE2 contents in the lung

**Figure 7:**
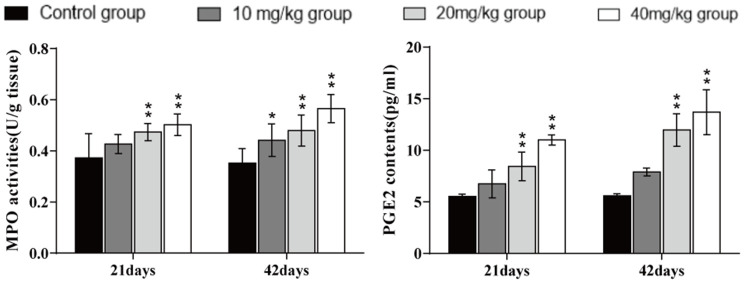
**Changes in MPO activities and PGE2 contents in the lung at 21 and 42 days of the experiment.** Data are presented with the mean± standard deviation (n=8). *p < 0.05, compared with the control group; **p < 0.01, compared with the control group.

The myeloperoxidase (MPO) activities and prostaglandin E2 (PGE2) contents were significantly higher (*p* < 0.01) in the 20 and 40 mg/kg groups at days 21 and 42 of the experiment than in the control group, as shown in Figure 7.

### Changes of mRNA expression and protein expression levels of NF-κB and IκB in the lung

**Figure 8:**
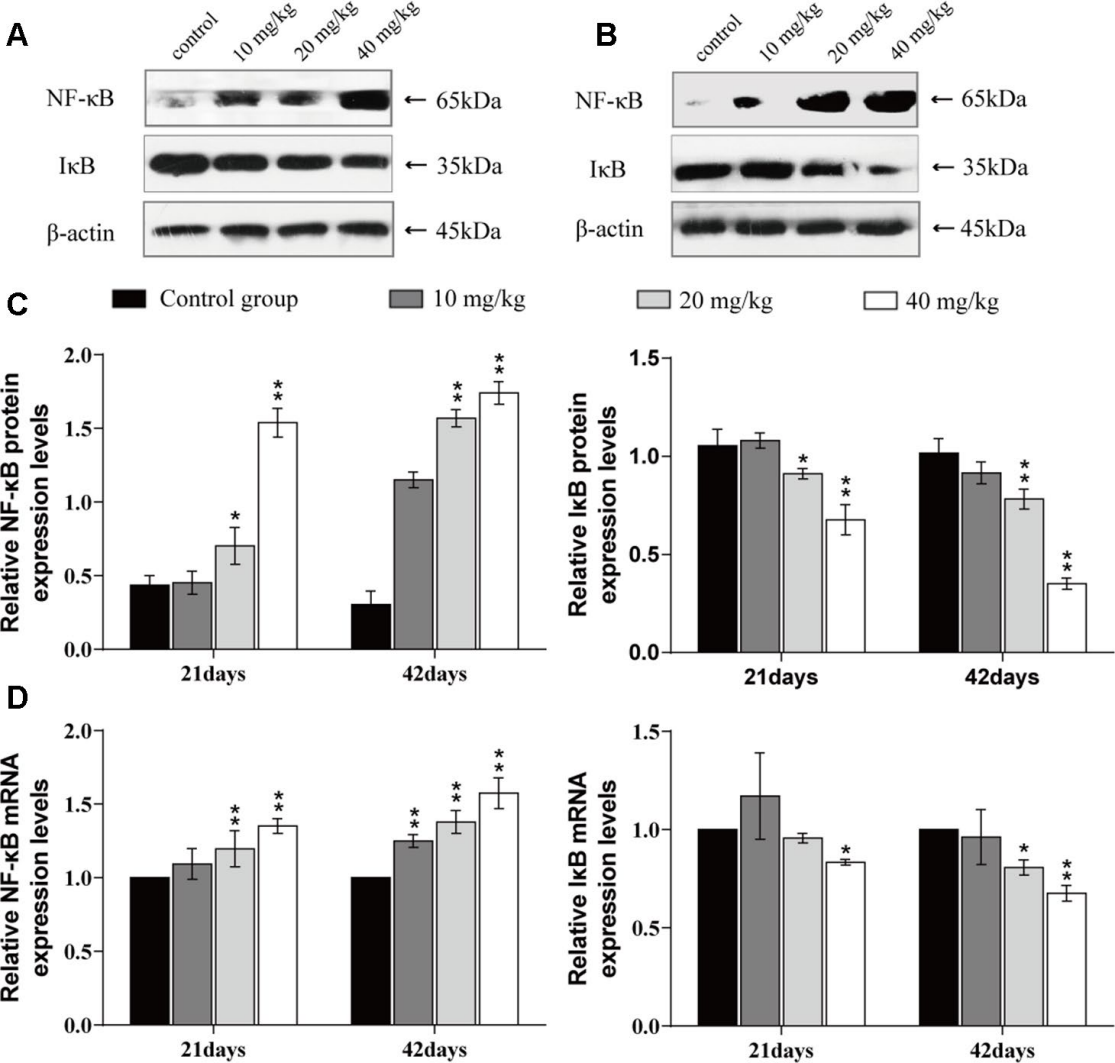
**Changes of mRNA expression and protein expression levels of NF-κB and IκB in the lung at 21 and 42 days of the experiment.** (**A**) Western blot assay of NF-κB and IκB at 21 days. (**B**) Western blot assay of NF-κB and IκB at 42 days. (**C**) The relative protein expression levels of NF-κB and IκB. (**D**) The relative mRNA expression levels of NF-κB and IκB. Data are presented with the mean± standard deviation (n=8). *p < 0.05, compared with the control group; **p < 0.01, compared with the control group.

The mRNA and protein expression levels of nuclear factor-kappa B (NF-κB) were significantly higher (*p* < 0.01 or *p* < 0.05) in the 20 and 40 mg/kg groups at 21 and 42 days of the experiment than in the control group. The mRNA and protein expression levels of inhibitory kappa B (IκB) were significantly decreased (*p* < 0.01 or *p* < 0.05) in the 20 and 40 mg/kg groups at 21 and 42 days when compared to the control group. The results are shown in Figure 8.

### Changes in mRNA expression and protein expression levels of inflammatory cytokines in the lung

The mRNA expression levels and protein expression levels of cyclooxygenase-2 (COX-2), interleukin-1β (IL-1β), interleukin-8 (IL-8), and interleukin-6 (IL-6) were significantly increased (*p* < 0.01 or *p* < 0.05) in the 20 and 40 mg/kg groups in comparison to the control group at 21 and 42 days of the experiment.

**Figure 9:**
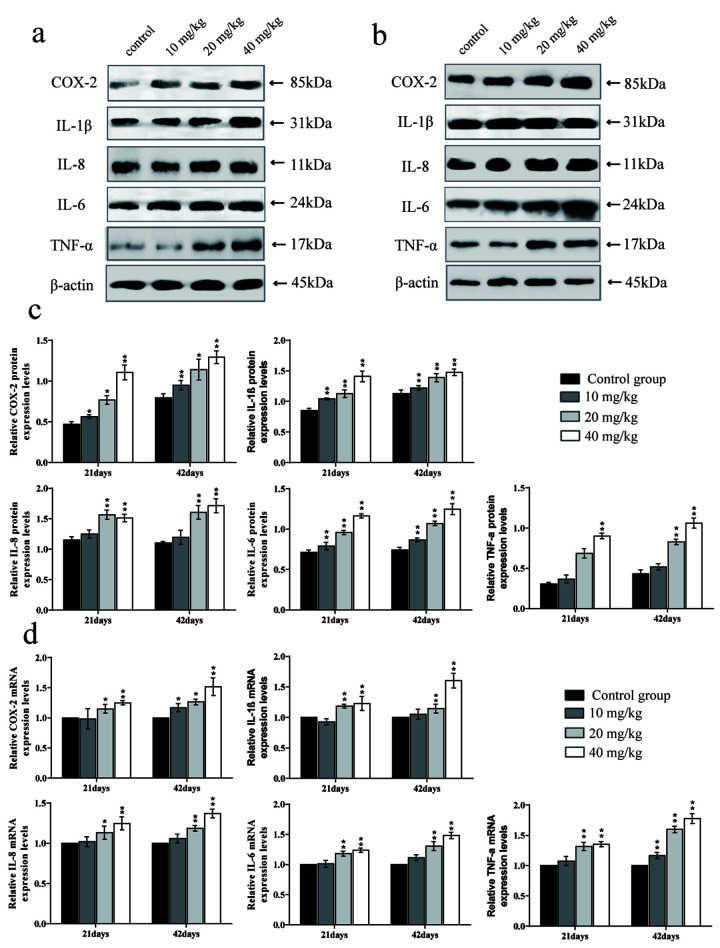
**Changes of mRNA expression levels and protein expression levels of pro-inflammatory cytokines in the lung at 21 and 42 days of the experiment.** (**A**) Western blot assay of pro-inflammatory cytokines at 21 days. (**B**) Western blot assay of pro-inflammatory cytokines at 42 days. (**C**) The relative protein expression levels of pro-inflammatory cytokines. (**D**) The relative mRNA expression levels of pro-inflammatory cytokines. Data are presented with the mean± standard deviation (n=8). *p < 0.05, compared with the control group; **p < 0.01, compared with the control group.

**Figure 10:**
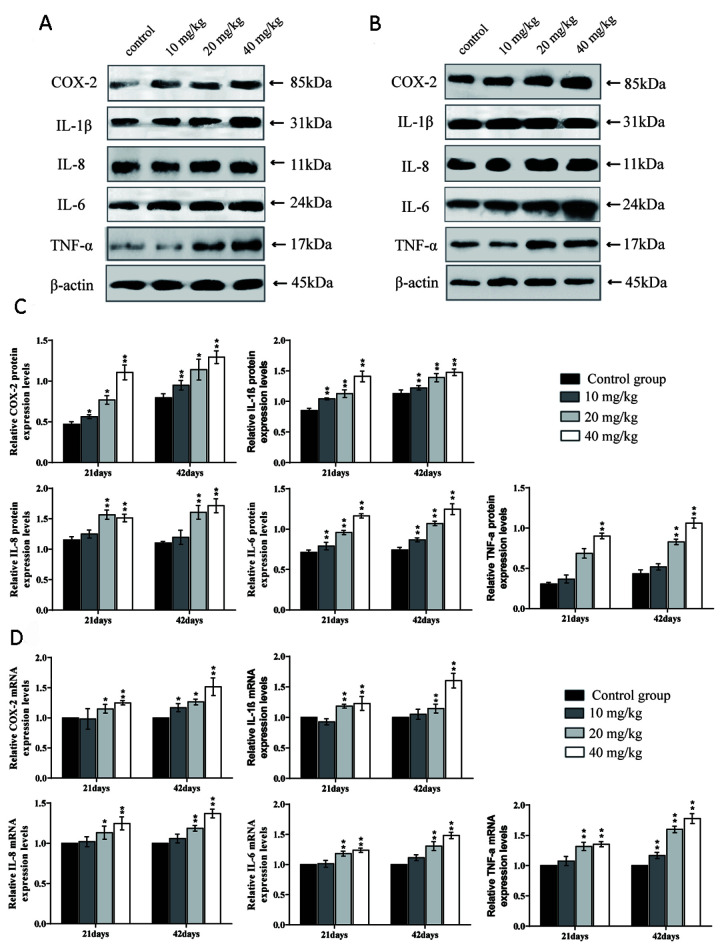
**Changes of mRNA expression levels and protein expression levels of anti-inflammatory cytokines in the lung at 21 and 42 days of the experiment.** (**A**) Western blot assay of anti-inflammatory cytokines at 21 days. (**B**) Western blot assay of anti-inflammatory cytokines at 42 days. (**C**) The relative protein expression levels of anti-inflammatory cytokines. (**D**) The relative mRNA expression levels of anti-inflammatory cytokines. Data are presented with the mean± standard deviation (n=8). *p < 0.05, compared with the control group; **p < 0.01, compared with the control group.

The mRNA and protein expression levels of tumour necrosis factor-α (TNF-α) were significantly higher (*p* < 0.01) in the 40 mg/kg group at 21 days of the experiment and in the 20 and 40 mg/kg groups at 42 days than in the control group. The mRNA expression levels and protein expression levels of interleukin-2 (IL-2), interleukin-4 (IL-4), and interleukin-10 (IL-10) were significantly decreased (*p* < 0.01 or *p* < 0.05) in the 20 and 40 mg/kg groups at 21 days and in the three CuSO_4_-treated groups at 42 days when compared to in the control group. The results are shown in Figures 9 and 10.

## DISCUSSION

In summary, this study firstly found that CuSO_4_ could induce oxidative stress, apoptosis, and inflammatory responses in the mouse lung.

We observed histopathological changes induced by CuSO_4_, alveolar walls were thickened in varying degrees with the dose- and time-dependent lesions, which mainly due to inflammatory cells infiltration and capillary congestion. Also, the pulmonary injuries were strongly consistent with the accumulation of Cu in the lung. It has been suggested that one possible molecular mechanism involved in the Cu toxicity is the formation of ROS, which leads to lesions via oxidative damage [55]. Our results showed that CuSO_4_ induced the production of ROS in the pulmonary tissue, and then excessive ROS caused oxidative damage to DNA (increased 8-OHdG contents) and lipid peroxidation (increased MDA contents). To reveal causes of the increased ROS, we tested the ASA and AHR abilities. The results showed that ASA and AHR abilities were reduced, which indicated that CuSO_4_ decreased the capacity of the lung to scavenge ROS and disturbed the dynamic balance between ROS production and elimination of ROS. Once the antioxidant function is impaired, free radicals (including oxygen free radicals, hydroxyl free radicals and nitrogen free radicals, *etc*.) are accumulated, which then causes oxidative stress in the body [56]. Kubbw et al. has found that rats consuming the zinc-deficient (0.5 mg/kg), high-copper (over 15 mg/kg) diet have the lowest weight gain and have an increase in endogenous free radical production in lung [57]. We also found an increase in the NO contents, iNOS enzyme activities, and mRNA expression levels, leading to oxidative damage in the lung. NO is an important nitrogen free radical in the body, which can also induce inflammation [58].

The increased ROS may indicate that the functioning of the antioxidant defence system is weakened [59], including decreased antioxidant enzymes (SOD, CAT, and GSH-PX) activities and non-enzymatic scavenger (GSH) content. SOD and CAT are important antioxidant enzymes that play a major role in ROS clearance [60]. GSH is an important intracellular antioxidant in alveolar epithelial cells [61]. GSH-dependent enzymes such as GSH-PX can remove hydrogen peroxide by oxidising GSH to GSSG, but ROS can oxidise GSH into GSSG, too much GSSG will weaken the protective effect of GSH, and the dysfunction of GSH will also aggravate the damage to organs [62, 63]. In the present study, the results showed that CuSO_4_ decreased CAT, SOD, and GSH-Px activities, GSH contents, and also the GSH/GSSG ratio was decreased in the lung, which were consistent with the report that copper can enhance oxidative stress, reduce the activities of antioxidant enzymes (SOD and CAT) and decrease the contents of GSH in brain tissues of chicken [51]. Cinar et al. (2014) has found that oral intake of CuSO_4_ can induce oxidative stress by reducing the activity of enzymes such as CAT and SOD in broiler serum [64]. Other studies have shown that the reduced intracellular GSH is associated with activation of NF-κB [65]. The further to explore the molecular mechanism of CuSO_4_-decreased antioxidant enzyme activities, we also detected mRNA expression levels of antioxidant enzymes in the lung. The results indicated that the mRNA expression levels of CuZnSOD, MnSOD, CAT, and GSH-PX were lower in the CuSO_4_-treated groups than in the control group, which were consistent with the reduction of these antioxidant enzyme activities. Decreased activities and mRNA expression levels of antioxidant enzymes promote oxidative stress, which plays critical roles in the pathogenesis of various diseases [66, 67].

In conclusion, our results showed that CuSO_4_ induces oxidative stress in the lung by promoting excessive ROS production and increased NO contents, and reducing antioxidant enzyme activities, GSH contents, and the GSH/GSSG ratio, which contribute to pulmonary lesions and dysfunction. Also, this study is the first to be focused on CuSO_4_-induced oxidative stress in the lung.

It has been known that ROS and oxidative stress play important roles in the early stages of apoptosis [68]. Next, we explored whether, or not, apoptosis was also involved in the mechanism of CuSO_4_-cuased toxicology in the lung. After CuSO_4_ treatment, the results indicated that the percentage of apoptosis was increased in the CuSO_4_-treated groups at 21 and 42 days. Apoptosis is a process controlled by multiple genes, such as bcl-2 family and caspase family, which are conserved among species [69]. As a group of important apoptotic regulators, the bcl-2 family can indirectly regulate caspases in relevant apoptotic pathways [70, 71]. Results in the present study showed that CuSO_4_ could cause a significant decrease in the protein expression levels and mRNA expression levels of anti-apoptotic proteins (Bcl-2 and Bcl-xL), while the protein and mRNA expression levels of pro-apoptotic proteins (Bax and Bak) were significantly increased in the lung. Hsien et al. (2008) has also observed that Cu-induced apoptosis was accompanied by the increased Bax and Bak expression levels and a decreased Bcl-2 expression level in neuroblastoma cells [72]. We also observed an increased Bax/Bcl-2 ratio in this research. Lee et al. (2008) has indicated that the increase of the ratio between Bax and Bcl-2 is the key point in the occurrence of apoptosis [73]. This increased Bax/Bcl-2 ratio leads to changes in mitochondrial membrane permeability and the release of apoptotic proteins, such as Cyt c [74]. In addition, excessive ROS production with decreasing concomitant in GSH contents also leads to mitochondrial membrane permeability and induction of apoptotic cell death in cultured cells [75]. Cyt c can cleave and activate caspase-9, which then activates downstream caspase-3 and cell apoptosis [76]. In this study, CuSO_4_ increased mRNA expression levels of caspase-9 and -3 and protein expression levels of cleaved-caspase-9 and -3, which contributed to apoptotic occurrence. The reports have also found that apoptosis is caused by copper-induced ROS formation in MCF7 cells [77], rat liver [78], and chicken intestine [79]. In summary, the present study was the first in which CuSO_4_-induced apoptosis was observed in the lung, and this was accompanied by decreasing Bcl-2, Bcl-xL mRNA expression levels and protein expression levels, and increasing Bax, Bak, caspase-3, and caspase-9 mRNA expression levels and protein expression levels, and the Bax/Bcl-2 ratio.

Inflammation is also a crucial toxicological mechanism of copper [80]. Myeloperoxidase (MPO) activity is used to measure the inflammatory degree in tissues and organs, and our results suggested that MPO activities were increased, which was consistent with the increased MPO arising from excessive accumulation of ROS [81]. This study is the first to investigate the mechanism of NF-κB activation in pulmonary inflammation by CuSO_4_ intake in mice. NF-κB is considered to be a major cellular transcription factor in inflammatory processes, and NF-κB activation has been identified as an important feature of inflammatory pulmonary diseases [82, 83]. Normally, NF-κB and IκB combine to form an inactive NF-κB-IκB complex [84]. In this study, The expression levels of IκB mRNA and protein were decreased, and the expression levels of NF-κB mRNA and NF-κB protein were increased, which demonstrated that CuSO_4_-induced IκB degradation promoted the activation of NF-κB. Concurrently, we observed that CuSO_4_ increased mRNA and protein expression levels of pro-inflammatory cytokines, such as TNF-α, COX-2, IL-6, IL-1β, and IL-8, which was in line with activation of the transcription factor NF-κB pathway. Our results are consistent with the report that copper can increase the expression levels of NF-κB, COX-2, IL-1β, TNF-α, IL-1β, and IL-8 mRNA and protein and exacerbate the damage and oxidative stress in zebrafish larvae tissues [85, 86].

It is well known that COX-2 is a classic proinflammatory cytokine, and plays an important role in the regulation of pulmonary inflammation [87]. As a downstream product of COX-2, PGE2 can not only induce the aggregation of inflammatory cells but also accelerate the process of peripheral inflammatory response induced by harmful stimuli [88]. In the present study, we observed that the PGE2 contents as well as the COX-2 mRNA and protein expression levels were significantly increased. Lu et al. (2008) has reported that intake of copper can up-regulate the expression of inflammation-related genes in the mouse brain, such as COX-2 and TNF-α [89]. iNOS, a proinflammatory marker in lung tissue, plays an essential role in exacerbating inflammation and can catalyse the production of NO, while excessive NO can increase the permeability of blood vessels and promote the infiltration of inflammatory cells [90–93]. In the present study, we found that CuSO_4_ increased the activities and mRNA expression levels of iNOS, and NO contents, which promoted lung inflammation.

On the contrary, IL-2, IL-4, and IL-10 are recognised as cytokines able to mediate immune suppression, which can inhibit the production of Th1 cells and reduce the release of pro-inflammatory cytokine [94, 95]. In the stage of acute pulmonary inflammation, IL-10 has significant anti-lymphocytes and neutrophilic infiltration [96]. Our results showed that CuSO_4_ decreased the mRNA and protein expression levels of IL-2, IL-4, and IL-10.

In conclusion, CuSO_4_ can increase MPO activities, activate the NF-κB pathway, and down-regulate anti-inflammatory cytokines, indicating that imbalance between pro-inflammatory and anti-inflammatory cytokine induces inflammatory responses in the lung. CuSO_4_-induced inflammatory responses contribute to pulmonary lesions and dysfunction.

## MATERIALS AND METHODS

### Experimental animals and diets

A total of 240 four-week-old ICR mice (half male and half female) obtained from the Chengdu Dossy Experimental Animals were used in the present study. The animals were housed in separate polypropylene cages, and diet and water were provided *ad libitum* throughout the experiment. The mice were fed a full-price diet provided by Dossy. After a week of rest and acclimatisation, mice were equally divided into four different groups (each with *n* = 60). The control group was given orally distilled water only, groups I, II, and III were given CuSO_4_ orally at the dose of 10, 20, and 40 mg/kg body mass, respectively. Mice were administered their respective doses daily by gavage for 42 consecutive days, and the gavage volume was 1 ml/100 g body mass.

The animal protocols and all procedures of the experiment were performed in compliance with the laws and guidelines of Sichuan Agricultural University Animal Care and Use Committee.

### Histopathological observation of the lung

At 21 and 42 days, eight mice (male female half) in each group were humanely killed and their lungs were removed, fixed in 4% paraformaldehyde solution, dehydrated with increasing concentrations of ethanol, cleared with xylene and embedded in paraffin. And then lungs were serial sectioned at 5μm thickness, stained with hematoxylin and eosin (H&E), and observed by optical microscopy.

### Determination of pulmonary Cu contents

At 42 days of the experiment, eight mice (male female half) in each group were humanely killed and their lungs were removed, weighed, dried, and collected for the determination of the Cu contents. The Cu contents in the lung were measured according to a reference method [97].

### Determination of the oxidative and anti-oxidative parameters in the lung

At 21and 42 days of age during the experiment, eight mice (male female half) in each group were humanely sacrificed, and the lungs were immediately stored at 4 °C cold phosphate buffer saline in a chilled homogeniser, and centrifuged at 3000 rpm for 15 min. Thereafter, the supernatant was transferred into new Eppendorf tubes. The commercial kits were purchased from Nanjing Jiancheng Bioengineering Institute (Nanjing, China) and used to detect ASA (Cat. No. A052-1) and AHR (Cat. No. A018), MDA (Cat. No. A003-1), CAT (Cat. No. A007-1), T-SOD (Cat. No. A001-1), GSH-Px (Cat. No. A005), and GSH (Cat. No. A061-1) according to the manufacturer’s instructions. Pulmonary 8-OHdG levels were measured using ELISA according to the manufacturer’s instructions.

### Detection of MPO, iNOS activities, and NO and PGE_2_ contents

At 21 and 42 days into the experiment, eight mice (half male, half female) in each group were humanely sacrificed, and the lungs were immediately stored at 4 °C cold phosphate buffer saline in a chilled homogeniser, and centrifuged at 3500 rpm for 10 min. Thereafter, the supernatant was transferred into new Eppendorf tubes. The commercial kits were purchased from Nanjing Jiancheng Bioengineering Institute (Nanjing, China) and used to detect MPO (Cat. No. A044), iNOS (Cat. No. A014-1), and NO (Cat. No. A013-2) according to the manufacturer’s instructions. Pulmonary PGE_2_ levels were measured using ELISA according to the manufacturer’s instructions.

### Apoptosis and ROS analysis by flow cytometry

At 21and 42 days, eight mice (half male, half female) in each group were humanely sacrificed, and the lungs were immediately stored at 4 °C phosphate buffer saline, and then were cut up to make a cell suspension, which was filtered through a 350-mesh nylon screen. The cells were washed twice with ice-cold phosphate buffer saline (PBS, pH 7.2-7.4), and then suspended in PBS at a concentration of 1 × 10^6^ cells/ml.

Thereafter, a total of 100 μL cell suspension were transfer into a 5 mL culture tube and centrifuged at 800*g* for 5 min for apoptosis testing. PE Annexin V and 7-aminoactinomycin (7-AAd) staining were used to dye the specimens. The mixture was gently shaken and then left in the dark for 15 minutes. Then 400 μl binding buffer was added to the tube, and the apoptosis rate of lung cells was detected and analysed by flow cytometry (FACS Calibur, BD, USA). The results were analysed using the Mod Fit LT for Mac V3.0 computer program.

Some 300 μL of the aforementioned cell suspensions were taken and transferred to another centrifuge tube, and stained with 10 μM DCFH- DA for 20 min at 37 °C for ROS testing. Then the cells were washed with PBS and centrifuged (600*g*, 5 min) once more. The supernatant was discarded, and cells were resuspended in 0.5 ml PBS and counted by flow cytometry (FACS Calibur, BD, USA).

### Determination of mRNA expression levels of antioxidant enzymes, inflammatory cytokines, and apoptotic proteins in the lung by qRT-PCR

At 21 and 42 days, lungs of eight mice in each group (half male, half female) were removed, stored in liquid nitrogen, and then Total RNA was extracted with the RNAiso Plus, and reverse transcribed into cDNA by using the Prim-Script™ RT reagent Kit as per the manufacture’s specification. The gene sequences of CuZnSOD, MnSOD, CAT, GSH-PX, Caspase 3, Caspase 9, Bax, Bak, Bcl-2, Bcl-xL, NF-κB, IκB, COX-2, IL-1β, IL-8, IL-6, and TNF-α IL-2, IL-4 and IL-10 were retrieved from NCBI, and the primers of these genes (Tables 1–3) were synthesised by Sangon Biotech (Shanghai, China). β-actin of mice was chosen as the reference gene. qRT-PCR reaction conducted on a C1000 Thermal Cycler (BIO RAD, USA) by using the SYBR^®^ Premix Ex TaqII (Takara, China) in accordance with the standard steps. All data output from the qRT-PCR experiments were analysed using the 2^-ΔΔCT^ method.

**Table 1:** List of primers of the antioxidant enzymes in qRT-PCR analysis.

**Gene symbol**	**Accession number**	**Primer sequence (5'–3')**	**Product size**	**Tm(°C)**
CuZn- SOD	NM205064	F: CGCAGGTGCTCACTTTAATCC	119bp	57
		R: CTATTTCTACTTCTGCCACTCCTCC		
Mn-SOD	NM204211	F: CACTCTTCCTGACCTGCCTTACG	146bp	57
		R: TTGCCAGCGCCTCTTTGTATT		
CAT	NM001031215	F: CTGTTGCTGGAGAATCTGGGTC	160bp	61
		R: TGGCTATGGATGAAGGATGGAA		
GSH-Px	NM001277853	F: TTGTAAACATCAGGGGCAAA	140bp	61
		R: TGGGCCAAGATCTTTCTGTAA		

**Table 2:** List of primers of the apoptotic genes in qRT-PCR analysis.

**Gene symbol**	**Accession number**	**Primer sequence (5′–3′)**	**Product size**	**Tm (°C)**
caspase-3	NM_009810	F: ACATGGGAGCAAGTCAGTGG	149bp	60
		R: CGTCCACATCCGTACCAGAG		
caspase-9	NM_015733	F: GAGGTGAAGAACGACCTGAC	103bp	57
		R: AGAGGATGACCACCACAAAG		
Bax	NM_007527	F: ATGCGTCCACCAAGAAGC	163bp	61
		R: CAGTTGAAGTTGCCATCAGC		
Bak	NM_007523	F: CGCTACGACACAGAGTTCCA	175bp	60
		R: CACGCTGGTAGACGTACAGG		
Bcl-2	NM_009741	F: AGCCTGAGAGCAACCCAAT	159bp	59
		R: AGCGACGAGAGAAGTCATCC		
Bcl-xL	NM_009743	F: TGTGGATCTCTACGGGAACA	117bp	59
		R: AAGAGTGAGCCCAGCAGAAC		

**Table 3:** List of primers of the inflammatory mediators in qRT-PCR analysis.

**Gene symbol**	**Accession number**	**Primer sequence (5′–3′)**	**Product size**	**Tm (°C)**
NF-κB	NM205134	F: CTGAAACTACTGATTGCTGCTGGA	179bp	62
		R: GCTATGTGAAGAGGCGTTGTGC		
IκB	NM204588	F:TGAGGACGAGGACGATAAGC	146bp	58.8
		R: ACAACGTGATCGCCATTACCTG		
COX-2	NM001167718	F: CTTAAATTGAGACTTCGCAAGGATG	165bp	62
		R: TGGGACCAAGCCAAACACCT		
IL-1β	Y15006	F: CAGCCTCAGCGAAGAGACCTT	106bp	60
		R: CACTGTGGTGTGCTCAGAATCC		
IL-8	HM179639	F: CTGGCCCTCCTCCTGGTT	105bp	60
		R: GCAGCTCATTCCCCATCTTTAC		
IL-6	NM001314054.1	F:ACAAAGCCAGAGTCCTTCAGAG	86bp	60
		R:GCCACTCCTTCTGTGACTCC		
TNF-α	NM204267	F: CCCCTACCCTGTCCCACAA	100bp	58
		R: TGAGTACTGCGGAGGGTTCAT		
IL-2	NM_001303244.1	F: TGTGGAATGGCGTCTCTGTC	125bp	60
		R: AGTTCAATGGGCAGGGTCTC		
IL-4	NM_021283.2	F: ATGGATGTGCCAAACGTCCT	78bp	60
		R: AAGCACCTTGGAAGCCCTAC		
IL-10	NM_010548.2	F: TGCCTGCTCTTACTGACTGG	79bp	60
		R: CTGGGAAGTGGGTGCAGTTAT		
β-actin	NM_007393	F:GCTGTGCTATGTTGCTCTAG	117bp	60.9
		R:CGCTCGTTGCCAATAGTG		

### Determination of protein expression levels of apoptotic proteins and inflammatory cytokines in the lung by Western blot assay

At 21 and 42 days, lungs of eight mice in each group (half male, half female) were removed, stored in liquid nitrogen, and then homogenised in liquid nitrogen by using a mortar and pestle. The total protein in each sample was carried out using RIPA lysis buffer, and protein contents of lungs were measured by using the BCA Protein Assay kit. Then the protein samples were separated by sodium dodecyl sulphate-polyacrylamide gel electrophoresis (SDS-PAGE) (10%-15% gels), and protein standards were used as molecular weight marker. After electrophoresis, proteins were transferred to nitrocellulose filter membranes. The membranes were blocked with 5 % non-fat-dried milk in phosphate-buffered saline with 0.1 % Tween 20 (PBST) for 1 h. After primary antibodies were incubated overnight at 4 °C, the membranes were washed three times with PBS for 10 min and incubated with biotin-conjugated secondary antibodies for 1 h with gentle shaking and washed again with PBST. Blots were visualised by ECLTM (Bio-Rad, Hercules, CA, USA) and X-ray film. The detected indicators were: cleaved-caspase-3, cleaved-caspase-9, Bax, Bak, Bcl-2, Bcl-xL, NF-κB, IκB, COX-2, IL-1β, IL-8, IL-6, TNF-α, IL-2, IL-4, and IL-10.

### Statistical analysis

The significance of difference was analyzed by the SPSS version 17.0. The results were shown as means ± standard deviation. The analysis was performed with the one-way analysis of variance (ANOVA). The differences between control and experimental group(s) at *p* < 0.05 were considered significant.
